# Editorial: Advancements in plant omics for tackling biotic and abiotic stresses

**DOI:** 10.3389/fpls.2023.1208218

**Published:** 2023-05-29

**Authors:** Mudassar Nawaz Khan, Abu Hena Mostafa Kamal, Xiaojian Yin

**Affiliations:** ^1^Department of Biotechnology & Genetic Engineering, Hazara University, Mansehra, Pakistan; ^2^Metabolomics Core, Baylor College of Medicine, Houston, TX, United States; ^3^State Key Laboratory of Natural Medicines, Institute of Pharmaceutical Science, China Pharmaceutical University, Nanjing, China

**Keywords:** abiotic & biotic stress, omics, genomics, transcriptomics, proteomics, metabolomics, phenomics, plant

Climatic change is now an established fact that poses a threat to the growth and yield of many economically important crops. The effects of climate change are seriously affecting human food production. As a result of the increasing global population, these threats require a great deal of attention so that effective strategies can be designed to handle the worsening scenarios.

With the recent advancements in omics sciences, such as genomics, transcriptomics, proteomics, metabolomics, and phenomics, scientists are using modern technologies to reveal stress-response mechanisms in plants. Advanced omics-related technologies are being used to develop stress tolerance in economically important crops. With the transfer of omics technologies to plant breeders, new stress-resistant plant varieties are being developed. Omics-based approaches are thus revolutionizing efforts to develop stress-tolerant crops.

The goal of the proposed Research Topic “*Advancements in plant omics for tackling biotic and abiotic stresses*” was to provide an interactive platform for scientists across the world to cluster their recent research regarding the application of advanced omics approaches to reveal plant stress-response mechanisms and develop biotic and abiotic stress resistance in plants. Based on recent omics advancements, various researchers report their findings in this Research Topic.

From the published manuscripts in this section, we can draw the conclusion that omics-based approaches are frequently being used as powerful tools to identify stress- related genes in all kinds of plant species. Chromosome localization, segmental duplication, and collinearity of cold stress- related bZIP transcription factors (LchibZIPs) were identified using a genome-wide approach in *Liriodendron chinense* (Li et al.). Furthermore, potential new roles of LchibZIPs were identified through protein-protein interaction analysis.

The genome of *Magnolia hypoleuca* was constructed at a chromosomal level and the cold tolerance of this plant was explored based on the genome (Zhou et al.). The study reports that tandem and proximal duplicates experienced more rapid sequence divergence, and a more clustered distribution on chromosomes played a significant role in facilitating cold tolerance in *Magnolia hypoleuca*.

In a study on soybean roots, the regulatory mechanism of low-phosphorus stress on phosphorus transport was explored using combined metabolomic, proteomic, and transcriptomic approaches (Li et al.). Low- phosphorus stress resulted in phosphorus allocation to UTP and glucose-6-phosphate. The frequency of glycolysis was increased, while the synthesis of malic acid was reduced to promote phosphorustransport in roots. The synthesis and transport of several sugars and organic phosphorus- containing compounds was inhibited under low- phosphorus stress.

T ranscriptomic analysis has been used to reveal the mechanism of response to chilling stress in *Lonicera japonica* (Zhang et al.). Among the differentially expressed genes, those relating to protein metabolism, transport, signaling, and secondary metabolism were upregulated. Chilling stress activated phenylpropanoid-flavonoid and carotenoid pathways in *L. japonica* leaves. This led to the elevation of calcium homeostasis and stimulation of brassinosteroid signaling, which in turn led to the regulation of phenylpropanoid-flavonoid/carotenoid- related transcription factors.

In a study on *Gossypium hirsutum* by Shuya et al., genomic techniques were employed to investigate the roles of *SAC* genes in ovule development and stress responses. A total of 157 *SAC* genes were identified in eight cotton species. Key genes related to the ovule, flower, and fiber development were identified. Drought and salinity treatment revealed differential expression of some of the *SAC* genes.

From these current research findings, it is evident that integrated omics approaches have taken the leading role in unraveling key mechanisms involved in plant stress responses. Moreover, further advancements in omics tools comprising genomics, transcriptomics, proteomics, and metabolomics will allow scientists to reveal complex mechanisms and identify the key factors associated with resistance to adverse climate change in economically important plants. It is also evident that integrated omics approaches are more revealing when they are applied to address complex biological questions.

## Author contributions

MNK being the editor opted for this Research Topic, made the concept paper and submitted it for the approval. He played the major role in all the events related to the Research Topic. AHMK and XY joined the editor and helped him in reviewing the manuscripts and writing the editorial. All authors contributed to the article and approved the submitted version.

